# A Keen Lad

**Published:** 1909-11

**Authors:** 


### A Keen Lad.

"I had always heard that New Englanders were 'smart.'" a young physician who has "graduated" from a village practice remarked the other day, "but hardly thought it developed at such an early age."

He smiled reminiscently, then continued:

"Just after I settled in Dobbs Corners a twelve-year-old boy called on me one evening.

"'Say, Doc. I guess I got measles,' he remarked, 'but nobody knows it 'cept the folks at home, and they ain't the kind that talks, if there's any good reason to keep quiet.'

"I was puzzled, and I suppose I looked it.

"'Aw; get wise, Doc,' my small visitor suggested. 'What will you give me to go to school an' spread it among all the kids in the village?'"—*September Lippincott's.*

---

ALCOHOL used in the home dissipates and wastes the substance and material resources of the family; it tends to weaken and ultimately to overthrow the authority of the family to the great injury of the children; it opens the door of the home to the most vicious forms of self-indulgence and impurity; it is the implacable enemy of all that belongs to the ethical advance of the community.—*Mrs. Bramwell Booth at London Congress.*

---

MR. MANN, Dean of the Medical School of the University of Buffalo, New York, voices the sentiments of those who think alcohol unnecessary. He says: "I think the medical profession could get along perfectly well without the use of alcohol, except in the manufacture of drugs. I do not suppose I have used a pint of alcohol in the last ten years. I think the tendency of the medical profession throughout the country is to give up alcohol in the treatment of disease."—*Temperance Journal.*

Dr. Boris Sidis, of Harvard University, in an address before the Summer School on "The Education of Man's Genius" offered a rational suggestion when he said: "The final and most valuable result of education is the recognition of evil. Let the child understand what he should avoid and why. Don't hide the evil from him, but explain it; here is a most desirable course of training in education."

It is not necessary to drag the child through multitudinous repulsive details, but no education is just to him which does not warn him in advance of the exact dangers lying ahead in a course of action which affects life so radically as does the use of intoxicants and narcotics. If our supreme aim is the strengthening of character, it will not be reached by leaving the child in ignorance, but by giving him the truth and showing him how to avoid the pitfalls. Here is the true place of the so-called "positive" temperance instruction.—*Scientific Temperance Journal.*

---

The physician is entitled to prescribe anything under the blue canopy that he thinks may be of value to his patient, and it is an impertinence to ask him to do otherwise.—*Midland Druggist.*

---

When draining a deep-seated abscess found with an aspirator, do not remove the aspirating needle until the abscess has been freely opened. (It is allowable to replace the needle by a grooved director.)—*American Journal of Surgery.*

---

Hydrogen peroxide should not be injected into deep infections in loose areolar tissue, as the expanding gas pushes the infection into the uninfected areas. Its most useful field is in open places.—*American Journal of Surgery.*

---

In immobilizing the knee-joint the patient is more comfortable and better relaxation is secured if a very slight degree of flexion is maintained.—*American Journal of Surgery.*

---

The examination of the eye grounds will often be the first clue to a tumor of the brain.—*American Journal of Surgery.*

CARE OF THE MOUTH IN DIABETES MELLITUS.—Levison (*Detroit Medical Journal,* May, 1900, through *Therapeutic Gazette,* for September, 1909) points out that in the treatment of diabetes mellitus the care of the mouth is important. The organisms found in the buccal cavity thrive on the saccharine body fluids and a fetid breath, stomatitis, and pyorrhea alveolaris often result. Every diabetic patient should constantly and faithfully use a mouth wash, and of these the liquor antisepticus alkalinus [of the National Formulary] answers every requirement. The following prescriptions by Ortner are very useful:

℞ Beta naphthol ........................... gr. v
Sodium borate ........................... ℥i
Peppermint water ........................... ℥v
Distilled water ........................... Oii

M. et Sig.: Use several times daily as a mouth wash.

For stomatitis and painful gums the following is prescribed:

℞ Tincture of opium........................... ʒi

Potassium chlorate ........................... ʒiiss

Sodium borate ........................... ʒiiss

Orange flower water........................... ℥i

Distilled water ........................... Oii

M. et Sig.: Use as a gargle.

---

The absence of a "history" should never be allowed to weigh against the diagnosis of syphilis—especially hereditary and tertiary syphilis. The disease is often contracted unknowingly as well as innocently, as by nursing infants.—*American Journal of Surgery.*

